# Feasibility and Acceptability of a Palliative Care Intervention among Older Adults with Advanced CKD and Their Caregivers

**DOI:** 10.34067/KID.0000000622

**Published:** 2024-10-24

**Authors:** Fahad Saeed, Robert K. Horowitz, Rebecca J. Allen, Peggy Auinger, Ronald M. Epstein, Kevin A. Fiscella, Peter J. Veazie, Paul R. Duberstein

**Affiliations:** 1 Division of Nephrology, Division of Palliative Care, Department of Medicine, University of Rochester School of Medicine and Dentistry, Rochester, New York; 2 Department of Medicine, Division of Palliative Care, University of Rochester Medical Center, Rochester, NY; 3 Center for IT Engagement, Mount St. Joseph University, Cincinnati, Ohio; 4 Department of Neurology and Center for Health and Technology, University of Rochester Medical Center, Rochester, New York; 5 Department of Family Medicine and Center for Communication and Disparities Research, University of Rochester School of Medicine and Dentistry, Rochester, New York; 6 Department of Public Health, University of Rochester, Rochester, New York; 7 Department of Health Behavior, Society and Policy, Rutgers School of Public Health, Piscataway, New Jersey

**Keywords:** CKD, dialysis, geriatric nephrology, quality of life, palliative care, supportive care

## Abstract

**Key Points:**

- In the current pilot trial, palliative care (PC) consultation for older adults with advanced CKD was both feasible and acceptable.
- PC consultation was associated with significant improvements in the decision-making process for kidney therapy and in quality of life.
- A large-scale PC-based decision-support intervention for older adults with CKD aimed at examining person-centered outcomes is warranted.

**Background:**

In non-nephrology settings, specialty palliative care (PC) improves decision making, patient's quality of life (QoL), advance care planning, and certain indicators of the quality of end-of-life (EoL) care. This pilot randomized control trial (RCT) explored the feasibility and acceptability of a PC intervention, CKD-EDU, for adults aged 75 years and older with eGFR ≤25 ml/min and their caregivers.

**Methods:**

Participants randomized to the control group received standard nephrology care and routine kidney therapy education, whereas those randomized to CKD-EDU received a decision aid and met with a PC clinician up to three times to discuss kidney therapy decisions and EoL planning. Patients were assessed at baseline, 4–6, 12–14, and 24–26 weeks. Main outcomes included intervention feasibility and acceptability, decision conflict, and patient QoL. The mediating effects of reduced decision conflict on improved QoL were explored, as were the effects of CKD-EDU on advance care planning, EoL treatment intensity, and 6-month hospitalization. Statistical analyses encompassed descriptive analyses, adjusted repeated-measure models, mediation analyses, and logistic regression models.

**Results:**

Among the 127 eligible patients screened, 58 (46%) consented: 30 were randomized to CKD-EDU and 28 to the control arm. All patients completed baseline assessments and 89% completed at least 1 intervention session (*n*=26/29), underscoring intervention adherence and feasibility. Similarly, assessment completion rates at 4 (83%, *n*=45/54), 12 (93%, *n*=42/45), and 24 (95%, *n*=40/42) weeks were high. The intervention received over 85% acceptability ratings for all questions. Patients exposed to CKD-EDU exhibited significant improvement in Decisional Conflict Scale scores (*P* = 0.003) at 4–6 weeks and improvements in QoL at 24–26 weeks (*P* = 0.02). Exploratory analyses were not statistically significant in this pilot study, but all effect sizes were in the predicted direction.

**Conclusions:**

This study demonstrates the feasibility and acceptability of CKD-EDU. A larger scale trial is warranted to assess its effectiveness in improving key outcomes important to patients and families.

**Clinical trial registry name and registration number:**

NCT03465449.

## Visual Abstract

**Figure:**
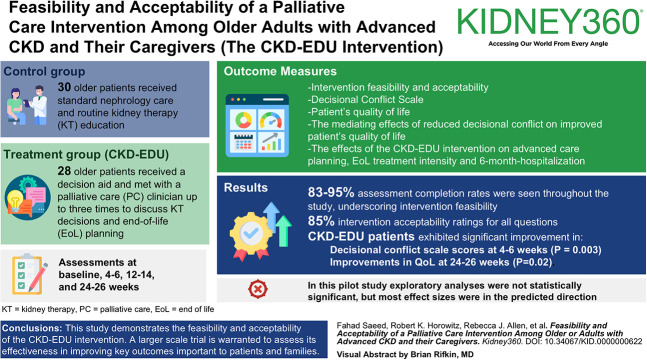

## Introduction

Older patients with advanced CKD often confront dialysis and other treatment decisions without adequate decisional support.^1–3^ They frequently report a lack of voice and respect for their choice in kidney therapy (KT) decision making.^1,4^ Dialysis is the default treatment option in the United States,^5^ but may not align with patients' goals because many patients prioritize quality of life (QoL) and functional independence over longevity.^6–8^ Dialysis regret is common,^9^ necessitating tailored interventions to address KT decisional needs.

Research in nonrenal patient populations considering burdensome or invasive treatments has shown specialist palliative care (PC) as beneficial—PC is associated with enhanced decision satisfaction, better QoL, more advance care planning (ACP), and reduced intensity of treatments at the end of life (EoL).^10–13^ However, the feasibility and acceptability of PC interventions for individuals aged 75 years and older remains unexplored.

To address the literature gap, we conducted a pilot randomized control trial (RCT) of a PC intervention, the CKD-EDU trial, focusing on feasibility and acceptability for both adults aged 75 years and older with advanced CKD and their caregivers. To generate effect sizes, we examined the preliminary effects of the intervention on KT decision making and QoL. We hypothesized that the CKD-EDU intervention would enhance QoL by reducing decisional conflict,^14^ and we investigated CKD-EDU's effect on ACP, EoL treatment intensity, and 6-month hospitalization rates.

## Methods

### Overview

The CKD-EDU study (2018–2021) was a pragmatic pilot RCT. The study protocol was approved by the institutional review board and was registered on ClinicalTrials.gov (NCT03465449) on July 3, 2018. Nephrologists, patients, and caregivers were recruited from outpatient nephrology clinics in Rochester, New York. All provided written consent to participate.

### Sample, Inclusion Criteria, and Recruitment

#### Recruitment of Nephrologists

To recruit nephrologists, the principal investigator (PI) and research coordinator (RC) outlined the eligibility criteria (Supplemental Table 1) at a faculty meeting. After the PI's departure to ensure anonymity, the RC obtained nephrologists' consent. Those absent from the meeting were approached individually by the RC, either through email or in person, to explain the study and obtain written consent for participation.

#### Recruitment of Patients

The RC identified eligible patients of participating nephrologists through electronic medical records (EMRs) on the basis of inclusion criteria (Supplemental Table 1). Then, the RC confirmed with nephrologists that patients required KT decision making likely because of progression to ESKD. The RC then approached eligible patients in person or by phone to explain the study and obtain written informed consent.

#### Recruitment of Caregivers

The RC asked consenting patients to list up to three caregivers in a ranked order. The RC then contacted the highest-ranked caregiver, either in person or by phone, to explain the study and obtain written informed consent. While patients could participate in the study without a caregiver (Supplemental Table 1), caregivers were not eligible to participate alone.

#### Randomization

Patients were randomly assigned to either the intervention or usual care arm using a random number table developed by a data analyst blinded to patient identities. The RC informed the participants and caregivers of their allocation status either in person or by phone after completing baseline assessments.

### Description of the PC Intervention

All patients continued routine care with their nephrologists. Control patients received KT education from the routine clinic educator, while intervention patients were mailed two booklets approximately 2 weeks before their PC visit: one on KT options and one a question prompt list for KT and EoL decisions. Just before the PC visit, the RC asked patients about their hopes, goals, and fears about KT. The RC recorded this information on a form and provided to the PC physician-interventionists, who then delivered the intervention in accordance with the National Consensus Project for Quality Palliative Care guidelines (see Supplemental Table 2).^15^ Notably, the intervention did not aim to persuade participants toward choosing a particular treatment option, but to help them explore their goals and understand the pros and cons of available KT options. PC physicians used a semistructured template to document visit details in the EMR and shared their notes with the patients' clinical teams.

#### PC Interventionists

Two PC physicians, including the PI, delivered the intervention using a training manual developed during the study. The second interventionist was trained using this manual through self-paced reading, an hour of in-person coaching, and a 10-minute phone call.

#### PC Intervention Dose

Patients, and their caregivers (if available), met with the PC interventionist for up to three sessions at patient–physician discretion, spaced 4–6 weeks apart. The first session lasted up to 60 minutes, and subsequent ones lasted approximately 30 minutes.

#### Usual Care Arm

Patients in the usual care arm received KT education per usual clinical routines.

#### Timing of Assessments

The unblinded RC administered surveys orally at baseline, 4–6, 12–14, and 24–26 weeks. Mortality surveillance continued until September 2022. The RC abstracted utilization data from the EMR.

### Assessments

#### Primary Outcomes

The main outcomes were the feasibility and acceptability of the CKD-EDU intervention. Feasibility was gauged by the success of patient recruitment, randomization, intervention retention, and completion of assessments by surviving patients. Acceptability was assessed using the Acceptability of the Intervention measure^16^ for both patients and caregivers. In addition, end-of-study interviews with patients, caregivers, and nephrologists provided qualitative insights, to be presented separately.

#### Secondary Outcomes

The secondary outcomes encompassed both decisional and QoL outcomes.

##### Decisional Conflict Scale

Assessments at baseline, 4–6, 12–14, and 24–26 weeks evaluated decision-making processes for both patients and caregivers.^17–19^ Patients used a ten-item scale with a ten-point Likert scale (0–10) allowing scores from 0 to 100. Caregivers completed a nine-item scale allowing scores from 0 to 90. Higher scores indicated greater decisional conflict and poor decision-making process.

##### Decisional Engagement Scale

Assessments at 4–6, 12–14, and 24–26 weeks measured CKD awareness, disease acceptance, decision self-efficacy, decision involvement, information seeking, and KT planning.^20^ This ten-item scale used a ten-point Likert scale (0–10) with scores ranging from 0 to 100, with higher scores indicating higher decisional engagement.

##### Modified Chronic Hemodialysis Knowledge Survey

Treatment decision making requires knowledge of the disease. This 21-item scale was administered at baseline and 12–14 weeks to evaluate patients' knowledge of KT options. Higher scores indicate greater knowledge.^21^

##### Decisional Regret Scale

This scale is assessed at 12–14 and 24–26 weeks (patients) and 24–26 weeks (caregivers) to measure regret levels for both groups.^22^ Using an eight-item scale with ratings on a five-point Likert scale (0–4), items were reverse coded as needed such that a higher number indicated more regret. For consistency with other 0–100 scales, scores were converted to a 100-point scale. Final scores were obtained by averaging items, with higher scores indicating greater regret.

##### Prognostic Concordance Questionnaire

Treatment decision making requires knowledge of prognosis. Prognostic concordance was determined by comparing patient and physician responses to three questions regarding survival probability and disease progression.^23^ These questions asked perceived likelihood of survival for 1 year, survival for 2 years, and cure. Responses were ordinal (*i.e*., 100%, 90%, 75%, 50%, 25%, 10%, and 0%). Patients' and nephrologists' responses were considered concordant if their responses were identical or differed by no more than two categories (*e.g*., patients reported 10%, physicians reported 50%). The prognostic concordance outcome was binary, indicating concordance on all three items or not. Physician responses at baseline were compared with patient responses at two time periods, baseline and 12–14 weeks.

##### KT Choice

Patient choice for KT included branching options, such as (*1*) unsure, (*2*) conservative kidney management, and (*3*) dialysis (in-center hemodialysis, peritoneal dialysis, or home hemodialysis).

##### Kidney Disease QoL-36 Scale

This scale was assessed at baseline, 12–14 weeks, and 24–26 weeks to gauge patients' QoL.^24^ This scale comprises five subscales: Short Form-12 Physical and Mental Component Summaries, Burden of Kidney Disease, Symptoms and Problems of Kidney Disease, and Effects of Kidney Disease. The Physical and Mental Component Summaries were both scored on a T-score metric (mean=50, SD=10, in the US general population). The three kidney-targeted subscales were scored by linearly transforming all items to a possible range of 0–100 and then averaging the items in the subscale. Kidney Disease QoL-36 items (KDQoL-36) were scaled so that higher scores reflected better perceived health-related QoL.

##### Zarit Caregiving Burden Scale

Administered at baseline and 4–6, 12–14 and 24–26 weeks, this widely used caregiver burden scale consists of 22 items rated on a five-point Likert scale ranging from 0 (never) to 4 (nearly always); sum of scores range from 0 to 88.^25^ Higher scores indicate greater burden.

### Other Outcomes

#### ACP

Patients were asked about the presence of medical orders for life-sustaining treatments, living wills, and health care proxies at baseline and 12–14 weeks.

#### Hospitalizations

The patient-reported number of hospitalizations was recorded throughout the 6-month study period.

#### Intensity of EoL Care Treatments

An unblinded RC conducted a review of EMRs for deceased patients up to September 2022 to assess EoL care intensity during the last 30 days of life^26^ using the following commonly used indicators: cardiopulmonary resuscitation, ventilation/intubation, in-hospital death, receipt of dialysis, G-tube usage, and intensive care approach versus hospice care.

### Statistical Analyses

All statistical analyses were conducted using SAS v9.4. Baseline demographic and clinical characteristics were compared between intervention and control groups using *t* tests for continuous features and chi-squared or Fisher's exact tests for categorical features.

We used repeated-measures analysis of covariance models to assess postbaseline decisional and QoL outcomes. The outcome measure was expressed as the change from baseline at each subsequent visit. These models included the assigned group (control or intervention), age, sex, self-identified race, baseline outcome value, visit week (as a categorical factor), and the interaction between the visit week and assigned group. For outcomes without a baseline assessment (*e.g*., patient decisional engagement and decisional regret), comparisons of the mean change from the first assessment were made for each group. Caregiver decision regret was only assessed at the 24–26-week visit, and this time point was modeled directly. Standardized effect sizes (Cohen's d) and intervention effects (intervention mean change minus control mean change) with 95% confidence intervals were calculated at each follow-up time point for each continuous outcome measure.

Binary outcomes (hospitalization, ACP, choice of KT, and two-point prognostic concordance) were modeled using generalized linear models with a logit link function. The same framework as described above was applied, and odds ratios with 95% confidence intervals and transformed log odds ratios approximating Cohen's d as standardized effect sizes are reported.^27^

Logistic regression was used to examine the relationship between the intervention versus control groups and rates of receiving an intensive approach during the last 30 days of life for deceased patients (ten in the intervention group and 11 in the control group). In addition, Poisson regression was used to examine the relationship between the intervention and control groups and the number of intensive EoL care events during the last 30 days of life for this subset of patients. Incidence rate ratios with 95% confidence intervals are reported.

Mediation analysis was performed to quantify the extent by which improvements in the Decisional Conflict Scale (DCS) explained the causal effect of randomized treatment assignment on the KDQoL burden subscale. Randomized treatment assignment was the exposure variable, KDQoL at the 24–26-week assessment was the outcome of interest, and the DCS at the 4–6-week assessment was the mediator. Estimates of a bias-corrected bootstrap (1000 replicates) confidence interval were used to determine the significance of the percentage mediated by the DCS.

Statistical significance was set at a *P* value < 0.05. Results are based solely on actual responses without any imputation of missing values. Consistent with prior behavioral research, a Cohen's d of ≥0.2, which is considered at least a small effect size, was deemed promising in this pilot study.^28^

## Results

Overall, 58 patients (mean±SD eGFR 19.3 ml/min±4.8) and 35 caregivers were enrolled. Table 1 presents baseline characteristics of patients and caregivers in the intervention CKD-EDU and control groups. The baseline mean age was 82 years (SD=5) for patients and 69 years (SD=14) for caregivers. Thirty-one patients (53%) and 24 caregivers (69%) were female. No significant differences were observed between the groups.

**Table 1:** Baseline demographic and clinical characteristics of patients and caregivers

Characteristics	Patients (*n*=58)		*P* Value	Caregivers (*n*=35)		*P* Value
	Intervention (*n*=30)	Control (*n*=28)		Intervention (*n*=19)	Control (*n*=16)	
Age	82.7 (5.5)	81.9 (5.1)	0.59	65.4 (14.8)	73.0 (13.4)	0.12
Female, No. (%)	16 (53.3)	15 (53.6)	0.99	14 (73.7)	10 (62.5)	0.48
Hispanic, No. (%)	3 (10.0)	1 (3.6)	0.61	1 (5.3)	0 (0.0)	0.99
**Self-identified race, *n* (%)**			0.38			0.54
Black	6 (20.0)	6 (21.4)		4 (21.1)	3 (18.8)	
Other	2 (6.7)	0 (0.0)		0 (0.0)	1 (6.2)	
White	22 (73.3)	22 (78.6)		15 (78.9)	12 (75.0)	
Annual income ≤$50,000, No. (%)	18 (60.0)	16 (57.1)	0.83	11 (57.9)	8 (50.0)	0.64
Married, No. (%)	12 (40.0)	16 (57.1)	0.19	12 (63.2)	12 (75.0)	0.45
**QoL subscales**						
Effect of kidney disease	93.4 (9.0)	88.5 (16.3)	0.17	NA	NA	NA
Symptom	84.2 (13.1)	82.2 (14.4)	0.58	NA	NA	NA
Burden of kidney disease	79.2 (26.3)	73.0 (23.0)	0.35	NA	NA	NA
Mental health	52.4 (9.7)	51.8 (10.2)	0.83	NA	NA	NA
Physical health	36.1 (11.3)	37.0 (13.1)	0.77	NA	NA	NA
Knowledge of renal replacement therapy options	3.6 (2.8)	3.7 (2.8)	0.91	NA	NA	NA
DCS	63.3 (17.2)	67.4 (15.7)	0.34	38.2 (17.4)	49.9 (18.9)	0.07
Prognostic concordance within two points, No. (%)^a^	11 (42.3)	9 (40.9)	0.92	NA	NA	NA
Caregiver burden	NA	NA	NA	13.9 (12.5)	20.9 (13.0)	0.11

Results are mean (SD) unless otherwise specified. Higher patient quality-of-life subscale scores indicate better perceived health-related QoL; higher knowledge scores indicate greater knowledge; higher DCS scores indicate greater decisional conflict and poor decision-making process; higher caregiver burden scores indicate greater burden. DCS, Decisional Conflict Scale; NA, not available; QoL, quality of life.

a Prognostic concordance: *n*=26 intervention, *n*=22 control.

### Feasibility Outcomes

**Figure 1:**
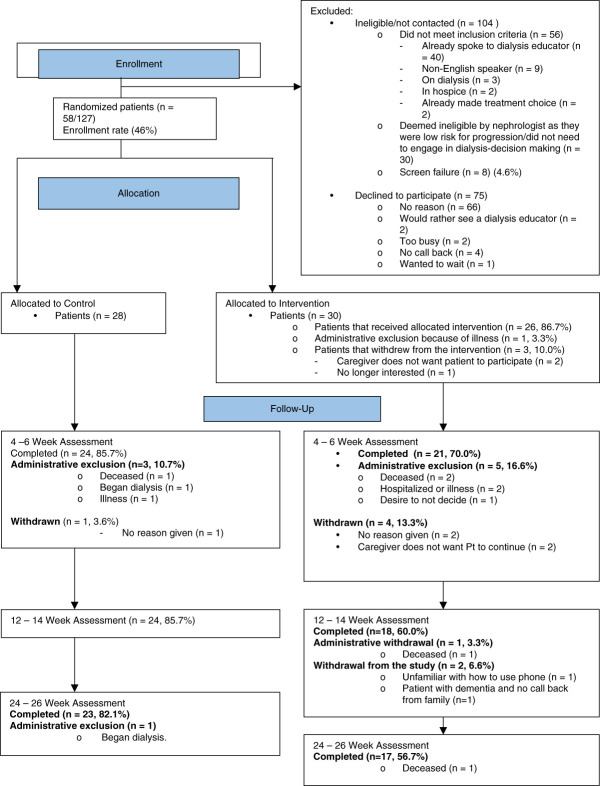
**CONSORT diagram for patient recruitment.** CONSORT, Consolidated Standards of Reporting Trials.

**Figure 2:**
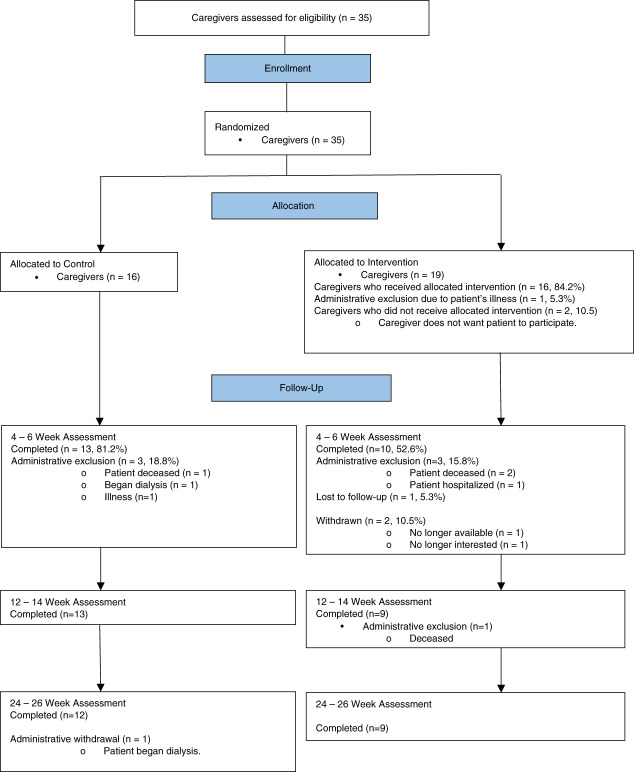
CONSORT diagram for caregiver recruitment.

Figures 1 and 2 provide a visual representation of patient and caregiver recruitment, retention, and randomization. Notably, demographic characteristics of patients deemed ineligible by nephrologists were not different from enrolled patients (data not shown), and all the approached nephrologists (*n*=28) were enrolled.

#### Patient Recruitment

Among the 231 screened patients, 127 (55%) were eligible, and among these, 58 consented to participate (enrollment rate of 44%).

#### Randomization

Of these 58 patients, 30 were randomly allocated to the intervention arm and 28 to the control arm. All randomized patients completed the baseline assessments, but one patient became ineligible because of acute illness. Twenty-six of the remaining 29 (89%) received the intervention. Three patients did not participate in the intervention, with two citing caregiver influences and one losing interest.

#### Retention

At the 4–6-week assessment, 45 of the 54 remaining patients (83%) completed the assessments. At the 12- to 14-week assessment, 42 of 45 patients (93%) completed the assessments. At 24–26 weeks, 40 of 42 patients (95%) completed the intervention (Figure 1). We have listed the reasons for dropouts in Figure 1.

#### Safety

No adverse effects were reported or observed.

### Acceptability

#### Patients

Twenty-one of 26 intervention participants completed the acceptability survey.^16^ The intervention received high acceptability ratings from patients, with 95% (*n*=20) feeling very comfortable or comfortable receiving care from a PC clinician, 100% (*n*=21) finding the PC physician very helpful or helpful in making decisions, and 86% (*n*=18) indicating a likelihood or high likelihood of recommending PC services to others.

#### Caregivers

All ten caregivers who completed the acceptability survey (100%) reported feeling very comfortable or comfortable receiving care from a PC clinician, found the PC physician to be very helpful or helpful in making decisions, and expressed a likelihood or high likelihood of recommending PC services to others.

### Early Evaluation of the Intervention's Effectiveness

Table 2 presents the findings of adjusted repeated-measures models related to decisional outcomes. Consistent with predictions, patients assigned to CKD-EDU experienced a greater improvement in the DCS^17^ from baseline to the 4–6-week assessment compared with the control group with a standardized effect size (d) of 1.0 (*P* = 0.003). Caregivers also experienced reduced decisional conflictfrom baseline to 4–6 weeks (d=1.3; *P* = 0.01). Nonsignificant decisional outcomes relate to patient Decisional Engagement Scale (d=0.5; *P* = 0.14), knowledge of renal replacement therapy options at 12–14 weeks (d=0.5; *P* = 0.17), and patient Decisional Regret Scale at 12–14 weeks (d=0.3; *P* = 0.43).

**Table 2:** Intervention effect on the basis of decisional conflict, regret, and knowledge

	Visit Mean (SEM)	Intervention Mean Change (95% CI) from Initial Visit	Visit Mean (SEM)	Control Mean Change (95% CI) from Initial Visit	Standardized Effect Size	Intervention Effect	*P* Value
**Patient DCS, wk**							
Baseline	64.3 (2.5)	—	64.3 (2.5)	—			
4–6	22.1 (5.3)	−42.2 (−53.0 to −31.5)	41.9 (5.6)	−22.3 (−33.8 to 10.9)	1.0	−19.9 (−32.2 to 12.5)^a^	0.003
12–14	44.1 (4.4)	−20.2 (−29.0 to −11.3)	51.1 (4.7)	−13.2 (−22.7 to −3.7)	0.5	−7.0 (−15.8 to 1.8)	0.12
24–26	44.4 (4.7)	−19.9 (−29.4 to −10.3)	47.6 (4.9)	−16.7 (−26.5 to −6.8)	0.2	−3.2 (−12.9 to 6.6)	0.51
**Patient Decisional Engagement Scale^b^, wk**							
4–6	80.6 (2.0)	—	80.6 (2.0)	—			
12–14	85.4 (3.5)	4.8 (−2.3 to 11.9)	80.6 (3.7)	−0.01 (−7.4 to 7.4)	0.5	4.8 (−1.7 to 11.3)	0.14
24–26	84.5 (4.0)	3.9 (−4.3 to 12.1)	83.9 (4.1)	3.3 (−5.0 to 11.6)	0.05	0.6 (−8.0 to 9.1)	0.89
**Knowledge of renal replacement therapy options, wk**							
Baseline	4.0 (0.4)	—	4.0 (0.4)	—			
12–14	4.6 (1.0)	0.6 (−1.4 to 2.7)	3.4 (1.1)	−0.6 (−2.8 to 1.6)	0.5	1.2 (−0.5 to 3.0)	0.17
**Patient Decisional Regret Scale^c^, wk**							
12–14	33.7 (3.4)	—	33.7 (3.4)	—			
24–26	25.6 (6.4)	−8.1 (−21.1 to 5.0)	20.9 (7.0)	−12.8 (−27.0 to 1.5)	0.3	4.7 (−7.2 to 16.5)	0.43
**Caregiver DCS, wk**							
Baseline	46.2 (3.9)	—	46.2 (3.9)	—			
4–6	11.5 (7.5)	−34.7 (−50.6 to −18.8)	28.1 (5.6)	−18.1 (−30.0 to −6.1)	1.3	−16.6 (−29.1 to −4.1)^a^	0.01
12–14	16.1 (8.4)	−30.1 (−47.8 to −12.4)	29.8 (6.4)	−16.4 (−30.0 to −2.8)	0.8	−13.7 (−29.9 to 2.5)	0.09
24–26	9.0 (7.4)	−37.2 (−52.8 to −21.6)	19.0 (5.6)	−27.2 (−39.2 to −15.3)	0.8	−9.9 (−22.4 to 2.6)	0.11
**Caregiver Decision Regret Scale^d^, wk**							
24–26	3.5 (12.0)	3.5 (−22.0 to 29.1)	18.6 (9.6)	18.6 (−1.8 to 39.1)	0.8	−15.1 (−33.7 to 3.5)	0.10

Results are initial and follow-up means (SEM) and estimates (95% CI) adjusted for intervention group, baseline score, age, sex, race, visit week, and visit week-by-intervention group interaction. Higher Decisional Conflict Scale scores indicate greater decisional conflict and poor decision-making process; higher DES scores indicate higher decisional engagement; higher knowledge scores indicate greater knowledge; higher Decision Regret Scale scores indicate greater regret. CI, confidence interval; DCS, Decisional Conflict Scale; SEM, standard error of the mean.

a *P* < 0.05.

b Comparison of mean change from 4 to 6 weeks adjusting for 4–6-week score.

c Comparison of mean change from 12 to 14 weeks adjusting for 12–14-week score.

d Comparison of mean scale at specific week (not change from baseline). Model does not adjust for baseline score.

We found that 35.3% of patients were unsure about their final choice in the intervention group compared with 52.2% of patients in the control group at 24–26 weeks (d=0.4; *P* = 0.33), and 59% of intervention patients shared two-point prognostic concordance with their nephrologist compared with 35% of controls at 12–14 weeks (d=0.3; *P* = 0.33).

Table 3 presents the findings of adjusted repeated-measures models related to QoL outcomes. We observed improvement in scores on the Burden of Kidney Disease Subscale^24^ (d=0.8; *P* = 0.02) and nonsignificant QoL findings (Table 3) in the Effects of Kidney Disease Subscale^24^ (d=0.4, *P* = 0.21) and physical domain of QoL subscale^24^ (d=0.3, *P* = 0.45).

**Table 3:** Intervention effect on the basis of quality-of-life outcomes

	Visit Mean (SEM)	Intervention Mean Change (95% CI) from Baseline	Visit Mean (SEM)	Control Mean Change (95% CI) from Baseline	Standardized Effect Size	Intervention Effect	*P* Value
**Effect of kidney disease, wk**							
Baseline	90.1 (2.1)	—	90.1 (2.1)	—			
12–14	90.2 (4.0)	0.1 (−8.0 to 8.2)	90.1 (4.0)	0.001 (−8.1 to 8.1)	0.01	0.1 (−8.5 to 8.7)	0.98
24–26	92.4 (3.2)	2.3 (−4.2 to 8.8)	88.7 (3.4)	−1.4 (−8.4 to 5.6)	0.4	3.7 (−2.1 to 9.5)	0.21
**Symptom, wk**							
Baseline	82.8 (1.8)	—	82.8 (1.8)	—			
12–14	84.8 (3.8)	2.0 (−5.6 to 9.7)	87.8 (4.1)	5.0 (−3.2 to 13.3)	0.3	−3.0 (−10.1 to 4.1)	0.39
24–26	83.6 (3.7)	0.8 (−6.7 to 8.3)	88.2 (4.0)	5.4 (−2.7 to 13.6)	0.5	−4.6 (−11.4 to 2.2)	0.18
**Burden of kidney disease, wk**							
Baseline	75.4 (3.4)	—	75.4 (3.4)	—			
12–14	70.0 (5.7)	−5.4 (−17.0 to 6.2)	67.1 (6.1)	−8.3 (−20.7 to 4.2)	0.2	2.9 (-7.4 to 13.2)	0.57
24–26	68.2 (5.9)	−7.2 (−19.3 to 4.8)	55.0 (6.3)	−20.4 (−33.2 to −7.6)	0.8	13.2 (1.9 to 24.5)^a^	0.02
**Mental health, wk**							
Baseline	52.1 (1.5)	—	52.1 (1.5)	—			
12–14	52.9 (2.2)	0.8 (−3.7 to 5.3)	55.9 (2.4)	3.8 (−0.9 to 8.6)	0.5	−3.0 (−7.2 to 1.1)	0.15
24–26	48.9 (2.8)	−3.2 (−8.8 to 2.4)	51.1 (2.8)	−1.0 (−6.5 to 4.6)	0.2	−2.2 (−8.3 to 3.9)	0.47
**Physical health, wk**							
Baseline	36.8 (1.9)	—	36.8 (1.9)	—			
12–14	37.2 (2.9)	0.4 (−5.4 to 6.2)	36.0 (3.0)	−0.8 (−6.9 to 5.2)	0.2	1.2 (−4.3 to 6.8)	0.65
24–26	37.9 (2.8)	1.1 (−4.6 to 6.9)	35.8 (3.0)	−1.0 (−7.0 to 5.1)	0.3	2.1 (−3.5 to 7.7)	0.45
**Caregiver burden, wk**							
Baseline	15.7 (2.5)	—	15.7 (2.5)	—			
4–6	18.9 (3.8)	3.2 (−4.9 to 11.2)	13.9 (3.3)	−1.8 (−8.8 to 5.2)	0.7	5.0 (−2.1 to 12.1)	0.16
12–14	13.3 (3.6)	−2.4 (−10.0 to 5.2)	15.0 (3.1)	−0.7 (−7.3 to 5.9)	0.3	−1.7 (−8.0 to 4.6)	0.58
24–26	14.7 (4.2)	−1.0 (−9.9 to 7.9)	14.4 (3.7)	−1.3 (−6.0 to 6.5)	0.02	0.2 (−8.5 to 9.0)	0.96

Results are baseline and follow-up means (SEM) and estimates (95% CI adjusted for intervention group, baseline score, age, sex, race, visit week, and visit week-by-intervention group interaction. Higher patient quality-of-life scale scores indicate better perceived health-related quality of life; higher caregiver burden scores indicate greater burden. CI, confidence interval; SEM, standard error of the mean.

a *P* < 0.05.

The observed improvement in DCS at 4–6 weeks did not demonstrate statistical significance in the mediation analyses for the Burden of Kidney Disease Subscale at 24–26 weeks (percentage mediated: 46%, bootstrap percentile 95% confidence interval: −2.7 to 140.3; *P* = 0.06).

Table 4 presents hospitalizations occurred in 18% of the intervention group compared with 35% of the control group (d=−0.6; *P* = 0.18). No ACP was reported in 5.6% of the intervention group compared with 16.7% of the control group (d=−1.0; *P* = 0.10). Whereas 4 of 11 control patients who died before September 2022 received intensive EoL treatments, only 1 of 10 intervention patients received intensive EoL treatments (incidence rate ratio 0.63 [CI, 0.31 to 1.28]; *P* = 0.2).

**Table 4:** Odds ratios (95% confidence interval) for intervention versus control for hospitalizations, ACP, MOLST and health care proxy

	Intervention, %	Control, %	Odds Ratio Intervention versus Control	*P* Value	Standardized Effect Size^a^	Adjusted Odds Ratio Intervention versus Control	*P* Value
Any hospitalizations at 24–26 wk	17.7	34.8	0.3 (0.1 to 1.5)	0.15	0.6	0.3 (0.1 to 1.7)	0.18
No ACP at 12–14 wk	5.6	16.7	0.2 (0.02 to 2.2)	0.20	1.0	0.2 (0.02 to 1.4)	0.10
No MOLST nor health care proxy at 12–14 wk	11.1	16.7	0.5 (0.1 to 3.2)	0.48	0.6	0.4 (0.1 to 2.3)	0.28

Results are odds ratios (95% confidence interval) on the basis of models with intervention group, visit week, and visit week-by-intervention group interaction. Adjusted models also include baseline age and sex (models did not converge when race was included). ACP, advanced care planning; MOLST, medical orders for life-sustaining treatments.

a On the basis of transformation of the log odds ratio to approximate Cohen's d effect size.

## Discussion

In this pilot study, we demonstrated the feasibility and acceptability of a novel PC intervention, CKD-EDU, for a cohort of individuals aged 75 years and older with advanced CKD contemplating KT choices. The encouraging effect sizes on all decisional outcomes, many QoL outcomes, ACP, and health care utilization outcomes warrant further investigation.

The findings on recruitment and retention suggest that conducting a PC trial among older adults with advanced CKD and caregivers is feasible. In addition, the intervention received high acceptance rates from both patients and caregivers. These findings generally align with the high acceptability of other PC-based interventions in patients with kidney disease and nonkidney populations,^29–31^ but are novel in the context of KT decision making for older people with CKD. To enhance participant retention, we plan to deploy additional strategies to improve patient retention in our future trial. These strategies may include providing patients with the choice to complete only key assessments, including an orientation session, involving caregivers to emphasize the significance of early engagement in the decision-making process, enhancing communication frequency of research staff with the participants (birthday cards, *etc*.), scheduling all assessment appointments up front, and using brief assessment batteries when possible.

Some significant findings related to the intervention's effect on improving the KT decision-making process and enhancing QoL suggest potential advantages associated with the multicomponent PC intervention. Comparable improvement in DCS scores have been documented in a large-scale trial assessing the effectiveness of an online decision aid.^18^ In addition, in our pilot study, improvements in the decision-making process were accompanied by better patient decisional engagement or activation^17^ and subsequent improvement in the patient's QoL. We expected that the intervention would lead to improved QoL by decreasing decisional conflict. However, the observed mediating effect was not statistically significant, although the effect was in the predicted direction. Effect sizes of CKD-EDU on knowledge of renal replacement therapy options scale (d=0.5), patient's Decisional Regret Scale at 12–14 weeks (d=0.03), decisional uncertainty (d=0.4), prognostic concordance (d=0.3), Effect of Kidney Disease subscale (d=0.4), Physical Health Subscale (d=0.3), caregiving burden at 12–14 weeks (d=−0.3), lack of ACP (d=1), and health care utilization (d=0.6) were in the predicted direction, although nonsignificant. Prior research has highlighted numerous positive effects of PC in nonrenal populations.^10–13^ Our study calls for a larger study to examine these outcomes adequately.

Our study demonstrates several strengths, limitations, and future research implications. As the first study exploring the feasibility, acceptability, and initial evaluation of the effectiveness of a PC intervention for aiding KT and EoL decision making in older adults, it provided valuable pilot data on intervention design (*e.g*., not limiting intervention sessions to three), timing of assessments, and use of validated DCS to assess major health decisions.^17–19^ Although not designed for hypothesis testing, the observed effect sizes for exploratory analyses are encouraging. While the findings may not be fully generalizable, high acceptability rates, feasibility, the encouraging effect sizes, the lessons learned, and the study manual will inform the development of larger scale PC interventions for older adults with advanced CKD and their caregivers. It is also plausible that lessons learned from PC interventions can inform future KT decision-making trials deploying nephrologists, dialysis educators, nurses, advanced nurse practitioners, social workers, or psychologists as interventionists delivering primary PC skills.

In conclusion, our study affirms the feasibility and acceptability of a PC intervention targeting adults aged 75 years and older who face decisions regarding KT and EoL choices. The study findings can guide the future large-scale evaluation of CKD-EDU for older adults with CKD.

## Supplementary Material

## Data Availability

All data are included in the manuscript and/or supporting information.

## Supplemental Material

Supplemental Table 1. Inclusion and exclusion criteria.

Supplemental Table 2. Key features of the palliative care visits highlighted in the study visit note template.
