# Supplementary material for: Feasibility and Acceptability of a Palliative Care Intervention among Older Adults with Advanced CKD and Their Caregivers
Source: Kidney360. 2024 Oct 24;6(2):236–46. doi: 10.34067/KID.0000000622 (PMC11882250; doi:10.34067/KID.0000000622)
Supplement: Supplementary file 1 [file kidney360-6-236-s001.pdf]

ASN American Society of Nephrology | JASN Journal of the American Society of Nephrology | KIDNEY360 Accessing Our World From Every Angle | CJASN Clinical Journal of the American Society of Nephrology

ASN Journal Disclosure Form

As per ASN journal policy, I have disclosed any financial relationships or commitments I have held in the past 36 months as included below. I have listed my Current Employer below to indicate there is a relationship requiring disclosure. If no relationship exists, my Current Employer is not listed.

R. Allen reports the following:
Employer: Mount Saint Joseph University; and Other Interests or Relationships: 20th Annual CRN Continuing Education Meeting, National Kidney Foundation, paid speaker at annual meeting.

I understand that the information above will be published within the journal article, if accepted, and that failure to comply and/or to accurately and completely report the potential financial conflicts of interest could lead to the following: 1) Prior to publication, article rejection, or 2) Post-publication, sanctions ranging from, but not limited to, issuing a correction, reporting the inaccurate information to the authors' institution, banning authors from submitting work to ASN journals for varying lengths of time, and/or retraction of the published work.

Name: Rebecca Jane Allen
Manuscript ID: K360-2024-000236R1
Manuscript Title: Feasibility and Acceptability of a Palliative Care- Intervention Among Older or Adults Advanced CKD and their Caregivers
Date of Completion: September 30, 2024
Disclosure Updated Date: September 30, 2024

ASN American Society of Nephrology | JASN Journal of the American Society of Nephrology | KIDNEY360 Accessing Our World From Every Angle | CJASN Clinical Journal of the American Society of Nephrology

ASN Journal Disclosure Form

As per ASN journal policy, I have disclosed any financial relationships or commitments I have held in the past 36 months as included below. I have listed my Current Employer below to indicate there is a relationship requiring disclosure. If no relationship exists, my Current Employer is not listed.

P. Auinger reports the following:
Employer: University of Rochester

I understand that the information above will be published within the journal article, if accepted, and that failure to comply and/or to accurately and completely report the potential financial conflicts of interest could lead to the following: 1) Prior to publication, article rejection, or 2) Post-publication, sanctions ranging from, but not limited to, issuing a correction, reporting the inaccurate information to the authors' institution, banning authors from submitting work to ASN journals for varying lengths of time, and/or retraction of the published work.

Name: Peggy Auinger
Manuscript ID: K360-2024-000236R1
Manuscript Title: Feasibility and Acceptability of a Palliative Care- Intervention Among Older or Adults Advanced CKD and their Caregivers
Date of Completion: September 30, 2024
Disclosure Updated Date: September 30, 2024

ASN
American Society of Nephrology
JASN
Journal of the American Society of Nephrology
KIDNEY360
Accessing Our World From Every Angle
CJASN
Clinical Journal of the American Society of Nephrology

ASN Journal Disclosure Form

Date: 9/4/2024

Author: Paul R. Duberstein, PhD

Manuscript ID: K360-2024-000236R1

Manuscript Title: Feasibility and Acceptability of a Palliative Care- Intervention Among Older or Adults Advanced CKD and their Caregivers

Disclosure Statements

Employer: Rutgers University
Ownership Interest: N/A
Consultancy: N/A
Research Funding: N/A
Honoraria: N/A
Patents or Royalties: N/A
Advisory or Leadership Role: N/A
Speakers Bureau: N/A
Other Interests or Relationships: N/A

ASN
American Society of Nephrology
JASN
Journal of the American Society of Nephrology
KIDNEY360
Accessing Our World From Every Angle
CJASN
Clinical Journal of the American Society of Nephrology

ASN Journal Disclosure Form

As per ASN journal policy, I have disclosed any financial relationships or commitments I have held in the past 36 months as included below. I have listed my Current Employer below to indicate there is a relationship requiring disclosure. If no relationship exists, my Current Employer is not listed.

R. Epstein reports the following:
Employer: University of Rochester; and Research Funding: NIH.

I understand that the information above will be published within the journal article, if accepted, and that failure to comply and/or to accurately and completely report the potential financial conflicts of interest could lead to the following: 1) Prior to publication, article rejection, or 2) Post-publication, sanctions ranging from, but not limited to, issuing a correction, reporting the inaccurate information to the authors' institution, banning authors from submitting work to ASN journals for varying lengths of time, and/or retraction of the published work.

Name: Ronald M. Epstein
Manuscript ID: K360-2024-000236R1
Manuscript Title: Feasibility and Acceptability of a Palliative Care- Intervention Among Older or Adults Advanced CKD and their Caregivers
Date of Completion: September 4, 2024
Disclosure Updated Date: September 4, 2024

ASN American Society of Nephrology JASN Journal of the American Society of Nephrology KIDNEY360 Accessing Our World From Every Angle CJASN Clinical Journal of the American Society of Nephrology

ASN Journal Disclosure Form

As per ASN journal policy, I have disclosed any financial relationships or commitments I have held in the past 36 months as included below. I have listed my Current Employer below to indicate there is a relationship requiring disclosure. If no relationship exists, my Current Employer is not listed.

K. Fiscella reports the following:
Employer: URMC

I understand that the information above will be published within the journal article, if accepted, and that failure to comply and/or to accurately and completely report the potential financial conflicts of interest could lead to the following: 1) Prior to publication, article rejection, or 2) Post-publication, sanctions ranging from, but not limited to, issuing a correction, reporting the inaccurate information to the authors' institution, banning authors from submitting work to ASN journals for varying lengths of time, and/or retraction of the published work.

Name: Kevin Fiscella
Manuscript ID: K360-2024-000236R1
Manuscript Title: Feasibility and Acceptability of a Palliative Care- Intervention Among Older or Adults Advanced CKD and their Caregivers
Date of Completion: September 30, 2024
Disclosure Updated Date: September 30, 2024

ASN Journal Disclosure Form

As per ASN journal policy, I have disclosed any financial relationships or commitments I have held in the past 36 months as included below. I have listed my Current Employer below to indicate there is a relationship requiring disclosure. If no relationship exists, my Current Employer is not listed.

R. Horowitz has nothing to disclose.

I understand that the information above will be published within the journal article, if accepted, and that failure to comply and/or to accurately and completely report the potential financial conflicts of interest could lead to the following: 1) Prior to publication, article rejection, or 2) Post-publication, sanctions ranging from, but not limited to, issuing a correction, reporting the inaccurate information to the authors' institution, banning authors from submitting work to ASN journals for varying lengths of time, and/or retraction of the published work.

Name: Robert Horowitz
Manuscript ID: K360-2024-000236R1
Manuscript Title: Feasibility and Acceptability of a Palliative Care- Intervention Among Older or Adults Advanced CKD and their Caregivers
Date of Completion: September 4, 2024
Disclosure Updated Date: September 4, 2024

ASN American Society of Nephrology JASN Journal of the American Society of Nephrology KIDNEY360 Accessing Our World From Every Angle CJASN Clinical Journal of the American Society of Nephrology

ASN Journal Disclosure Form

As per ASN journal policy, I have disclosed any financial relationships or commitments I have held in the past 36 months as included below. I have listed my Current Employer below to indicate there is a relationship requiring disclosure. If no relationship exists, my Current Employer is not listed.

F. Saeed reports the following:
Employer: University of Rochester Medical Center; Consultancy: KJT group; Research Funding: NIDDK K-23; ASN career development award, Renal research institute; and Honoraria: KJT group medical advisory council.

I understand that the information above will be published within the journal article, if accepted, and that failure to comply and/or to accurately and completely report the potential financial conflicts of interest could lead to the following: 1) Prior to publication, article rejection, or 2) Post-publication, sanctions ranging from, but not limited to, issuing a correction, reporting the inaccurate information to the authors' institution, banning authors from submitting work to ASN journals for varying lengths of time, and/or retraction of the published work.

Name: Fahad Saeed
Manuscript ID: K360-2024-000236R1
Manuscript Title: Feasibility and Acceptability of a Palliative Care- Intervention Among Older or Adults Advanced CKD and their Caregivers
Date of Completion: September 4, 2024
Disclosure Updated Date: June 5, 2024

ASN American Society of Nephrology · JASN Journal of the American Society of Nephrology · KIDNEY360 Accessing Our World From Every Angle · CJASN Clinical Journal of the American Society of Nephrology

ASN Journal Disclosure Form

As per ASN journal policy, I have disclosed any financial relationships or commitments I have held in the past 36 months as included below. I have listed my Current Employer below to indicate there is a relationship requiring disclosure. If no relationship exists, my Current Employer is not listed.

P. Veazie has nothing to disclose.

I understand that the information above will be published within the journal article, if accepted, and that failure to comply and/or to accurately and completely report the potential financial conflicts of interest could lead to the following: 1) Prior to publication, article rejection, or 2) Post-publication, sanctions ranging from, but not limited to, issuing a correction, reporting the inaccurate information to the authors' institution, banning authors from submitting work to ASN journals for varying lengths of time, and/or retraction of the published work.

Name: Peter J. Veazie
Manuscript ID: K360-2024-000236R1
Manuscript Title: Feasibility and Acceptability of a Palliative Care- Intervention Among Older or Adults Advanced CKD and their Caregivers
Date of Completion: October 7, 2024
Disclosure Updated Date: May 17, 2024
