# Supplementary material for: Feasibility and Acceptability of a Palliative Care Intervention among Older Adults with Advanced CKD and Their Caregivers
Source: Kidney360. 2024 Oct 24;6(2):236–46. doi: 10.34067/KID.0000000622 (PMC11882250; doi:10.34067/KID.0000000622)
Supplement: Supplementary file 2 [file kidney360-6-236-s002.pdf]

Supplemental Table 1. Inclusion and Exclusion Criteria

	Physicians	Patients	Caregivers
**Inclusion Criteria**	**1**.Member of Strong nephrology group working at Strong Memorial Hospital<br>**2.**Treat patients with CKD	**1.**Age ≥75 years old<br>**2.** GFR ≤ 25 ml/min)<br>**3**.Patient's nephrologist is enrolled in the study, and has seen that nephrologist at least once<br>**4**.Speaks English<br>**5.**Have not attended a dialysis education class or met with the dialysis education coordinator.<br>6. Have not made a dialysis decision	Consenting patients will be asked for permission to contact up to three caregivers, defined as a "family member, partner, friend or someone else who is involved with your health care issues, for example, someone who you talk to about personal issues including medical decisions or who comes to doctor appointments with you. This person may also help with routine day-to-day activities, like transportation or paperwork." The term "caregiver" is used here for scientific purposes only; it is not essential that this individual self-identify as a caregiver.<br>**1.**self-identified caregiver (per definition above)<br>**2.**21 years of age or older.<br>3.LAR of the patient
**Exclusion Criteria**	**1**.Expecting to leave in six months.	**1.**Patient has already been seen by a palliative care clinician or is enrolled in hospice<br>**2.** Is already on dialysis<br>**3.**Hospitalized at the time of recruitment	**1.**Support is offered primarily in a professional role (e.g., clergy).<br>**2.**Cognitive impairment

Supplemental Table 2. **Key features of the palliative care visits highlighted in the study visit note template**
Patients will receive a 60-minute initial visit, each follow up visits will be 30 minutes in duration.<br>1. Identifying goals, hopes and fears.<br>2. Listening to the illness narrative, conducting value affirmation exercise.[33,34,35]<br>3. Addressing kidney therapy knowledge needs (Teach Back Methodology).[36]<br>4. Discussing (ASK-TELL-ASK approach)[37] renal and overall prognosis after seeking patient permission.[38,39]<br>5. Firming up a kidney therapy decision that aligns with patient goals.<br>6. Engaging in end-of-life planning (Teach Back Methodology).[36]<br>7. Completing advance care planning documents<br>8. Providing anticipatory guidance—timing of dialysis initiation or referral to palliative care or discussion of prognosis.
